# Effects of vitamin D3 and its chemical analogs on the growth of Hodgkin’s lymphoma, in vitro

**DOI:** 10.1186/s13104-019-4241-0

**Published:** 2019-04-08

**Authors:** Rajendra Gharbaran, Bo Zhang, Luis Valerio, Onyekwere Onwumere, Madeline Wong, Jason Mighty, Stephen Redenti

**Affiliations:** 1 Department of Biological Sciences, Bronx Community College, The City University of New York, Bronx, NY 10453 USA; 2 Department of Biological Sciences, Lehman College, City University of New York, Bronx, NY 10468 USA; 3 Biology Doctoral Program, The Graduate School and University Center, City University of New York, 365 5th Avenue, New York, NY 10016 USA

**Keywords:** Vitamin D3, Hodgkin’s lymphoma, Cancer, Oncology, Calcitriol, EB1089, Calcipotriol, B cell malignancy

## Abstract

**Objective:**

Vitamin D receptor (VDR) activities have been noted for a number of B cell malignancies which showed varying sensitivities to vitamin D3 (1,25-dihydroxyvitamin D3, VD3, calcitriol) and its synthetic analogs. The objective of this study was to address the potential effects of VD3 and vitamin D3 analogs (VDAs) on the growth of Hodgkin’s lymphoma (HL), a malignant pathology of B cell origin, in vitro.

**Results:**

Immunofluorescence staining showed the expression of VDR by primary Hodgkin’s (H) and Reed–Sternberg (RS)—HRS-tumor cells in HL histological sections. Western blot analyses revealed expression of VDR in the HL cell lines Hs445, HDLM2, KMH2, and L428. One-way analysis of variance (ANOVA) on data obtained from water-soluble tetrazolium 1 (WST-1) cell proliferation assay showed decreased cell growth in HDLM2 and L428, 72 h after treatment with 10 µM of either VD3 of VDAs. Western blot analyses showed that treatment of L428 cells with the VDAs (calcipotriol and EB1089) resulted in modest increases in nuclear accumulation of VDR (nuVDR) compared to either dimethyl sulfoxide (DMSO) or VD3 treatments. nuVDR for DMSO control and VD3 was comparable. These results suggest that VD3 or VDAs may affect growth of HL.

## Introduction

Vitamin D3 receptor signaling and its therapeutic perturbation with vitamin D3 (VD3, 1,25-dihydroxyvitamin, calcitriol) or vitamin D3 analogs (VDAs) have attracted much attention in cancer therapies, including treatment of B cell malignancies [1]. This is largely due to reports that showed strong association between VD3 deficiency and increased risk of both solid and liquid tumors [2–5]. In subsets of B cell malignancies, low vitamin D is associated with worst prognosis—inferior event-free survival (EFS) and overall survival (OS) [2]. Studies showed that upon ligand (either VD3 or VDA) binding, VDR translocates to the nucleus where it modulates the expression of genes involved in cell growth and differentiation and apoptosis [6].

Hodgkin’s lymphoma (HL) is presented as a unique B cell neoplasm characterized by the presence of giant mononuclear Hodgkin’s (H) and multinuclear Reed–Sternberg (RS)—HRS-cells, which make up less than 1% of the cellular infiltrate of the tumor microenvironment. Although a previous immunohistochemical study showed that VDR is highly expressed in 80% of HL cases [7], very little is known about the influence of VD3/VDA–VDR interaction in this malignancy. Therefore, the goal of the current study was to investigate the effects of VD3/VDAs on the growth of HL via VDR expression, in vitro.

## Main text

### Methods and materials

#### Immunofluorescence

Immunofluorescence was performed as described by Gharbaran et al. [8] with some modifications. Tissue array (US Biomax) consisting of 5-μm thick formaldehyde-fixed paraffin-embed histological sections was dewaxed with 2× with xylene (10 min each), rehydrated in 100, 80, 70, 50% ethanol (5 min each), and rinsed briefly in 1× PBS. Antigen retrieval was performed by heating sections for 20 min in 10 mM Na citrate (pH 6.0) at 95 °C followed by cooling for 20 min at room temperature. Sections were subsequently blocked with BSA (5% in Tris buffered saline (TBS, Sigma) then incubated overnight with anti-VDR mouse monoclonal antibody (Santa cruz; sc-13133) diluted at 1:100 in blocking buffer, at 4 °C. The tissue array was next washed 3× in TBS for 5 min each. This was followed by incubation in Dylight 594-conjugated mouse secondary antibody (Jackson Immunoresearch) diluted at 1:500 in blocking buffer, in the dark for 1 h at room temperature, subsequent washes as described. The sections were covered with number 1 coverslips containing 10 µL of mounting medium containing DAPI (Vector Labs). Staining was visualized on a Nikon Ti Eclipse inverted microscope. Images were captured with a CoolSNAP HQ2 CCD camera (Photometric Scientific) coupled to the microscope and processed with Adobe Photoshop 7.0 (Adobe, San Jose, CA) for clarity. All steps were same for negative controls with the exception of incubation in primary antibody.

#### Cell lines and cell cultures

The human HRS-derived cell lines HDLM2, KMH2 and L428 were obtained from the German Collection of Microorganisms and Cell Cultures (DSMZ), Department of Human and Animal Cell Cultures, Braunschweig, Germany. Hs445 was obtained from the American Tissue and Cell Collection (ATCC). L428 and KMH2 were cultured in RPMI 1640 medium supplemented with 10% heat-inactivated fetal bovine serum (FBS) (Gibco BRL, Gaithersburg, MD), 1% l-glutamine, and penicillin/streptomycin. Culture media for HDLM2 and Hs445 were similar but contained 20% heat-inactivated FBS. All cells were maintained in a humid environment of 5% CO_2_ at 37 °C.

#### Vitamin D3 and its chemical analogs

VD3 or VDAs (calcipotriol and EB1089) were purchased from Cayman Chemicals and dissolved in DMSO to prepare a 10 mM stock which was aliquoted and stored at − 20 °C until ready to be used.

#### WST-1 cell proliferation assay

2 × 10^5^ cells/mL plated in 96-well plates overnight and treated with 10 μM each of VD3, calcipotriol, and EB1089 for 72 h were incubated in indicated culture conditions. Cell growth was assessed with WST-1 cell proliferation assay (Roche, USA), according to manufacturer’s instructions. In brief, 100 μL of treated cells were incubated with 10 µL of WST-1 reagent for 3 h following standard culture conditions. Absorbance was read at 450 nm on a Synergy H1 Hybrid microplate reader (BioTek). Cell growth (in percentages, %) was computed as ratio of absorbance (A450 nm) of treated cells relative to absorbance of control cells (DMSO) (A450 nm).

#### Preparation of nuclear extracts

L428 was plated at 1.5 × 10^5^ cells/mL in 60-mm petri and incubated overnight. Cells were then treated with 10 μM each of VD3 or VDAs or vehicle (DMSO) and incubated for another 48 h. Treated cells were harvested by centrifugation for 5 min at 300×*g*, then washed 2× with ice-cold 1× PBS. Cell membrane was lysed by resuspending the cells in 500 μL ice-cold hypotonic buffer (20 mM Tris–HCl, pH 7.4, 10 mM NaCl, 3 mM MgCl_2_) supplemented with Halt Protease and Phosphatase Inhibitor Cocktail (Thermofisher) and incubated on ice for 15 min, with occasional mixing. The lysate was then mixed with 25 μL of 10% NP_4_O by vortexing for 10 s. The homogenate was centrifuged at 3000 rpm for 10 min at 4 °C. Supernatant was transferred to a new tube and mix with RIPA (radioimmunoprecipitation) assay buffer (EMD Millipore) supplemented with protease and phosphatase inhibitor as described. Pellet (nuclear fraction) was resuspended in 150 μL ice-cold 1× RIPA buffer as described and incubated on ice, with vortexing at 10 min interval. Nuclear fraction was subsequently centrifuged for 30 min at 14,000×*g* at 4 °C and the supernatant aliquoted and stored at − 80 °C until ready for quantification by Bradford assay and analysis by sodium dodysulfate polyacrylamide (SDS-PAGE) gel and Western blot.

#### Preparation of total cell extracts

Treated cells were collected and rinse as described, then resuspended in ice-cold RIPA buffer supplemented with Halt Protease and Phosphatase Inhibitor Cocktail (Thermo Scientific, Rockford, IL, USA). Lysates were incubated for 5 min on ice with periodic mixing followed by centrifugation at 13,000×*g* for 10 min at 4 °C. Supernatant was transferred to new tube and stored at − 80 °C until ready for quantification by Bradford assay and SDS-PAGE and Western blot analyses.

#### Western blot

Protein concentrations were determined using Pierce^®^ BCA Protein Assay kit (Thermo Scientific) following the manufacturer’s instructions. Twenty micrograms of protein were then loaded per well. Proteins were separated on 10% SDS-PAGE and transferred to nitrocellulose membrane (Odyssey 0.22 µm) using Bio-Rad Mini-PROTEAN Electrophoresis System. The membrane was incubated overnight with the primary antibodies at the indicated dilution prepared in TBST containing 2% BSA: anti-VDR (Santa Cruz; sc-13133, 1:1000), anti-histone (Invitrogen; 24HC2, 1:2000), anti-α-tubulin (Sigma; T6074, 1:2000). After brief rinses with TBST, the membrane was incubated with IRDye^®^ 680RD Donkey anti-Mouse (LI-COR, 926-68072), and IRDye^®^ 800CW Donkey anti-Rabbit (LI-COR, 926-32213) for 30 min. Membrane was finally rinsed as described and protein signal was imaged using Odyssey-CLX imaging system (Li-COR).

#### Statistical analyses

Data analyses were performed using SAS 9.1.3, StatView 5, or JMP 4. Absorbance data reflection cell growth were presented as standard error of the mean (SEM) derived from triplicates. Analysis of variance (ANOVA) and F statistics were used to determine differences between the means as defined by p < 0.05. All experiments were repeated at least three times.

### Results

#### Expression of VDR

**Fig. 1:**
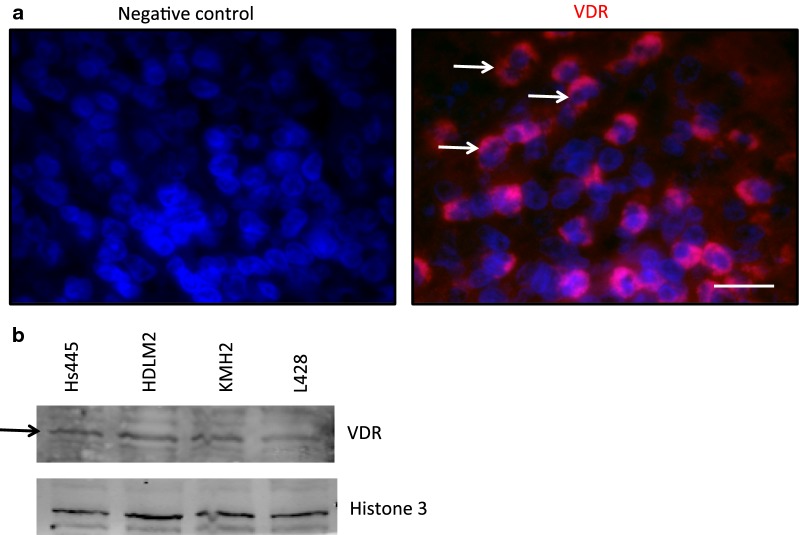
Detection of VDR expression in HL. **a** Tissue immunofluorescence staining showing the expression of VDR (red) in primary tumor cells of Hodgkin’s lymphoma, Hodgkin’s and Reed–Sternberg (HRS) cells. Nuclei of cells are stained with DAPI. Arrows indicate representative HRS cells. Scale bar = 100 μm. **b** Detection of VDR expression by Western blot in unstimulated HL cell lines. Arrow indicates VDR band. Histone was used as the house keeping protein

The physiological responses of VD3/VDAs are mediated via VDR. Therefore, we analyze HL tissue sections and cell lines for the expression of VDR. Our immunofluorescence staining showed the expression of VDR by HRS cells on tissue array (Fig. 1a). This observation is consistent with a previous immunohistochemical study which showed that VDR is expressed by almost 80% of HL cases [7]. VDR expression by HL cell lines was determined by Western blot analyses. Hs445, HDLM2, KMH2, and L428 showed relatively low expression but varying levels of VDR (Fig. 1b).

#### Effects of VD analogs on cell growth

**Fig. 2:**
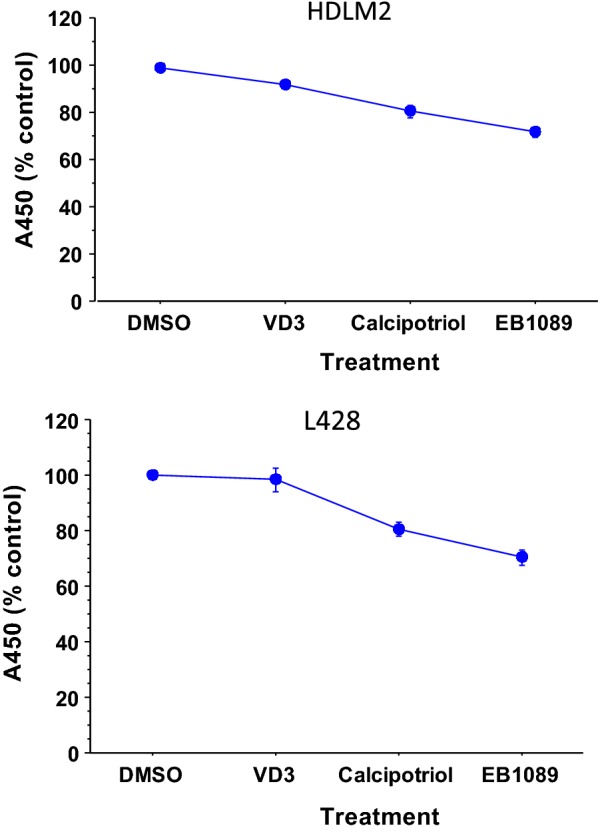
Effects of VD3 and VDAs on growth of Hodgkin’s lymphoma. L428 and HDLM2 cells treated with 10 μM of either VD3 or VDAs (calcipotriol and EB1089) for 72 h showed modest decrease in growth. EB1089 showed the strongest effect

Previous studies indicated growth-limiting activities of VD3/VDAs on both normal and malignant B lymphoid cells [9]. Therefore, we next studied the effects of VD3 and the VDAs calcipotriol and EB1089 on the growth of HL cells HDLM2 and L428. One-way ANOVA on data obtained from WST-1 cell proliferation assay revealed decreases in growth of cells treated with either VD3 or VDAs, after 72 h (Fig. 2). As expected, EB1089 showed a greater effect in reducing growth of both HDLM2 and L428.

#### Nuclear accumulation

**Fig. 3:**
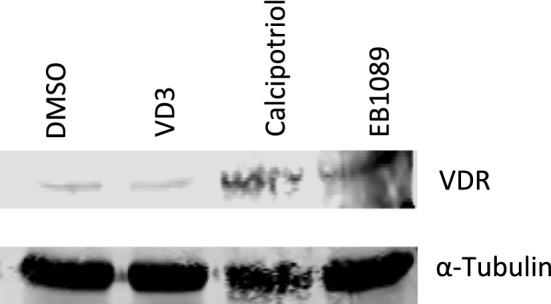
Nuclear accumulation of VDR in L428 after treatment with VD3/VDAs for 48 h. EB1089 and calcipotriol resulted in greater nuVDR. DMSO and VD3 showed comparable levels of nuVDR. α-tubulin was used as the housekeeping protein

It has been suggested that upon ligand (VD3/VDAs) binding, VDR translocates to the nucleus where it modulates the expression of target genes. Therefore, we tested the effects of VD3/VDAs on the nuclear accumulation of VDR by Western blot. L428 cells treated with 10 µM of VDAs for 48 h showed only a modest increase in nuVDR over either DMSO- or VD3-treated cells (DMSO and VD3 showed comparable effects on nuVDR)—Fig. 3. The modest changes in nuVDR in our study may be due to very low basal levels of VDR in unstimulated L428 cells.

### Discussion

Our data showed that VDR is expressed by HL cell lines and primary HRS cells. The HL cell lines used in this study showed low but varying levels of VDR expression. Also, expression of VDR by HRS cells is qualitatively in accordance with the study by Renne et al. [7]. The results also showed that VD3/VDAs reduced growth of HL, in vitro, 72 h after treatment. The reduced cell growth of L428 was associated with modest increases in nuVDR in response to treatment with calcipotriol and EB1089.

In our study, the dose of VD3 (10 µM) used to elicit only modest decrease in cell growth is considered to be at supra-physiological concentration and this may be a consequence of low basal levels of VDR expression in unstimulated cells. In follicular low grade non-Hodgkin’s lymphoma (NHL), low levels of VDR expression was detected in primary tumor cells and cell lines (SU-DHL4 and SU-DUL5), however high concentration of VD3 was used to produce growth restriction in vitro [10]. In contrast, cellular models of acute myeloid leukemia (HL-60 cells) and diffuse large B cell lymphomas (DOHH2, K422) express high levels of VDR and display moderate cytotoxicity and pro-apoptotic responses upon exposure to nanomolar concentrations of VD3 (and VDAs) [11]. These observations suggest the levels of VDR expression in unstimulated cells may influence extent of VD3 sensitivity of some B malignancies. Additionally, while VD3 fail to stimulate nuVDR in our study (compare to DMSO), nanomolar concentrations of VD3 resulted in significantly higher nuVDR in other B cell neoplasms [11].

Because low VDR expression may hinder anti-tumor activities of VD3, it will be important to develop strategies to induce increased VDR expression. One of these approaches involved ligand-stimulated upregulation of receptor. It has been reported that VD3 stimulation resulted in the VDR upregulation [12, 13]. However, VD3-stimulated upregulation may not be possible in all types of VDR-positive neoplasms. Other approaches include the use of a non-vitamin inducer. Progesterone directly induced upregulation of VDR expression in T lymphocytes, via binding progesterone receptor-binding elements in the intron region after the first noncoding exon of the VDR gene [14]. In addition, progesterone enhanced the anti-tumor activities of VD3 by upregulating of VDR expression in endometrial cancer cells [15]. Inducer-stimulation of VDR expression may be useful for cancer therapy in which primary tumor cells exhibit low levels of VDR expression.

Application of supra-physiological concentration of VD3 to suppress tumor growth in vivo can promote hypercalcemia and hypercalciuria [16], leading to hardening of soft tissues. As a result, low-calcemic VDAs were developed [11, 17]. Two of these molecules, calcipotriol and EB1089 showed anti-tumor activities in a number of studies [18, 19]. However, EB1089 appeared to be amongst the most potent anti-tumor VDA [20]. In our study, treatment with either calcipotriol or EB1089 resulted in significant decrease in cell growth which also correlated with greater nuVDR. Future studies are warranted to determine the underlying mechanism(s) by which calcipotriol and EB1089 limit growth of HL.

## Limitations

Our study has limitations that must be considered. A positive control such as the breast cancer cell line MCF7 or the myeloid leukemia cell HL-60, both of which previously showed significant sensitivities to VD3/VDAs were not included in our study. The current study also suffers from a lack of cell cycle analysis and other molecular details.

## Abbreviations

VDR: vitamin D receptor
VD3: vitamin D3
VDA: vitamin D3 analogs
HL: Hodgkin’s lymphoma
H: Hodgkin
RS: Reed–Sternberg
HRS: Hodgkin’s and Reed Sternberg
ANOVA: analysis of variance
nuVDR: nuclear accumulation of vitamin D receptor
DMSO: dimethyl sulfoxide
WST-1: water-soluble tetrazolium 1
EFS: event-free survival
OS: overall survival
FBS: fetal bovine serum
TBS: tris buffered saline
RIPA: radioimmunoprecipitation
SDS-PAGE: sodium dodysulfate polyacrylamide gel
BSA: bovine serum albumin
SEM: standard error of the mean
